# The Impact of Pentraxin 3 on Crohn’s Disease Phenotype

**DOI:** 10.3390/ijms252111544

**Published:** 2024-10-27

**Authors:** Anna Kofla-Dlubacz, Lilla Pawlik-Sobecka, Tomasz Pytrus, Agnieszka Borys-Iwanicka, Joanna Gorka-Dynysiewicz

**Affiliations:** 1 2nd Clinical Department of Paediatrics, Gastroenterology and Nutrition, Faculty of Medicine, Wroclaw Medical University, 50-367 Wroclaw, Poland; anna.kofla-dlubacz@umw.edu.pl (A.K.-D.); tomasz.pytrus@umw.edu.pl (T.P.); agnieszka.borys-iwanicka@umw.edu.pl (A.B.-I.); 2 Department of Basic Medical Sciences and Immunology, Division of Basic Medical Sciences, Faculty of Pharmacy, Wroclaw Medical University, 50-367 Wroclaw, Poland; 3 Department of Pharmaceutical Biochemistry, Faculty of Pharmacy, Wroclaw Medical University, 50-367 Wroclaw, Poland; joanna.gorka-dynysiewicz@umw.edu.pl

**Keywords:** pentraxin 3 [PTX3], phenotypes of Crohn’s disease, children’s gastrointestinal tract

## Abstract

Pentraxin 3 [PTX3] is an acute-phase protein playing an important role in the regulation of the humoral arm of immune response. As one of the molecules from the conservative family of pentraxins, PTX3 is a soluble mediator involved in the transduction of pro-inflammatory signals between immunocompetent cells. Additionally, recognizing damage-associated molecular patterns (DAMPs) during tissue injury mediates wound healing; therefore, its concentration potentially correlates with the severity of fibrosis. The aim of our study was to evaluate the value of the PTX3 measurement as a phenotypic marker of the stenotic form of Crohn’s disease. The research covered 63 patients, 35 with the narrowing type (B2) and 28 with the inflammatory type (B2) of CD. The mean concentrations of PTX3 in the study were as follows: 3.06 ng/mL (95% CI: 1.27–6.99) for the B1 phenotype, 4.89 ng/mL (95% CI: 2.98–13.65) for the B2 phenotype, and 3.04 ng/mL (95% CI: 1.01–4.97) for the control group. PTX3 concentrations reached the highest values in the B2 group and the lowest in the control group. The differences between the B1 and B2 groups were statistically significant at *p* < 0.001. The presented studies indicate the potential role of PTX3 in the monitoring of tissue remodeling and the development of fibrosis in CD.

## 1. Introduction

Inflammatory bowel diseases (IBDs) are a group of autoimmune disorders of the gastrointestinal tract that are induced by an imbalance in the immune response to stimulating environmental factors in genetically predisposed people [1].

The main entities, Crohn’s disease (CD) and ulcerative colitis (UC), although differing in their clinical course and predilection for spread in sections of the digestive tract, also have many common features, especially at the level of immune response abnormalities.

The regulation of the immune system takes place at many levels and, to date, a number of variations in the functioning of the signaling chain have been described, including innate and adaptive responses. The key elements are the integrity of the intestinal barrier, the loss of which leads to excessive exposure to the intestinal microbiome, and the promotion of pro-inflammatory factors of humoral and cellular-specific responses [2,3,4,5,6,7].

The identification of the inflammatory pathway IL12/23-IL12Rg1-SAT-JAK related to the activation of Th17 cells, which are the source of many further pro-inflammatory cytokines (IL17A, IL17F, IL22, IL26, chemokine CCL20), as well as the way activated lymphocytes migrate to the target site in the gastrointestinal tract, resulted in the development of therapeutic methods that are highly effective in the selective treatment of IBD. However, the complexity of mutual interactions between individual elements of the immune cascade makes it difficult to take full control over the course of the IBD and remains a challenge to overcome [8,9,10,11,12].

A particularly important and irreversible stage in the course of IBD is tissue remodeling associated with progressive fibrosis leading to the loss of function in the affected section of the digestive tract. It is typical of Crohn’s disease, where it leads to the development of intestinal strictures. However, the increased changeover of the extracellular matrix (ECM) is also described in the course of ulcerative colitis. In this process, mesenchymal cells including myofibroblasts, fibroblasts, and smooth muscle cells are stimulated by growth factors, including pro-inflammatory cytokines secreted by activated immune and epithelial cells along with intestinal antigens (pathogen-associated molecular patterns (PAMPs)), to produce matrix metalloproteinases (MMPs) and their inhibitors, which are known as TIMPs (tissue inhibitors of matrix metalloproteinases). These two groups of molecules take control over tissue remodeling and, in the case of MMP domination, result in the degradation of the extracellular matrix and chronic damage to the intestinal wall [13,14,15,16].

In this study, we aim to consider the role of molecules in transmitting signals into activation from external and internal antigens to cellular receptors on immune cells and their impact on further fibrosis. Soluble receptors of extracellular compartments, recognizing conserved antigenic patterns within invasive pathogens or PAMPs (pathogen-associated molecular pattern) and the damaged body’s own cells or DAMPs (damage-associated molecular patterns) are crucial for initiating and maintaining inflammation. These include, among others, pentraxins, which, by activating membrane Toll-like receptors (TLRs 1,2,4,5,6) and intracellular NOD receptors (nucleotide-binding oligomerization domain proteins, NOD1, NOD2) in myeloid, lymphoid, and vascular endothelial cells, are bridging factors of the adaptive immune response. The pentraxin family consists of two subgroups with different amino acid chain lengths: short pentraxins and long pentraxins (PTX 3, NP1, PTX 4) [17,18]. All pentraxins are soluble mediators that influence functions of the immune system and tissue remodeling.

Short pentraxins, including the acute-phase proteins PTX1 (CRP) and amyloid components PTX2 (SAP), are produced by the liver in response to bacterial and viral infections, and in case of the release of endogenous components of the cells, e.g., in the course of tissue necrosis or under the stimulation of IL-1 and IL-6, they are widely used in clinical practice as non-specific indicators of the intensity of the immune response. While CRP and SAP are classic acute-phase proteins, the role of long pentraxins is still under investigation.

Among long pentraxins, there are pentraxins 3 and 4 (PTX 3, PTX4) and the neuronal pentraxin 1(NP1).

Pentraxin 3 is a multimeric structure (a protein octamer with monomers connected by disulfide bonds) that, like short pentraxins, recognizes bacterial domains, apoptotic cells, amyloid fibers, and other endogenous particles of decaying cells. Its level increases strongly and quickly during inflammatory reactions, and what is particularly important in the development of autoimmune diseases is that it seems to be an inducer of the formation of autoantibodies found in autoimmune diseases, including autoantibodies against neutrophil cytoplasm (ANCA). The involvement of PTX3 has also been described in the process of tissue remodeling during fibrosis. The participation of PTX3 in the healing of tissue damage of the skin, liver, and lungs has been demonstrated in animal models. Its involvement in the remodeling of the extracellular matrix in humans has also been described [19,20].

These features make PTX3 an interesting marker potentially involved in initiating and maintaining the inflammatory process in inflammatory bowel disease [21]. The aim of this study is to evaluate the serum PTX 3 concentration in Crohn’s disease in the pediatric population.

## 2. Results

The control group consisted of 20 children aged 1 to 16.5 years old with functional disorders of the gastrointestinal tract, in whom organic disease was excluded during the diagnosis. The study group (n = 63 patients) aged 5 to 17.5 years was divided into two subgroups: B1 (n = 28 patients) aged 9 to 17.5 years and B2 (n = 35 patients) aged 5 to 17.5 years. The characteristics of the control and study groups are presented in Table 1 (Table 1). Both PTX3 and CRP levels were higher in the sera of patients from subgroup B1 (3.06 ng/mL, 95% Confidence Intervals (CI): 1.27–6.99 and 2.00 mg/L, 95% CI: 0.50–45.20, respectively) and subgroup B2 (4.89 ng/mL, 95% CI: 2.98–13.65 and 2.55 mg/L, 95% CI: 0.50–44.00) than in the control group (3.04 ng/mL, 95% CI: 1.01–4.97 and 0.5 mg/L, 95% CI: 0.5–4.30). In our studies, we observed higher levels of PTX3 in the fibrotic phase of Crohn’s disease compared to the inflammatory stage and the control group. The differences between the B1/B2 groups were statistically significant at *p* < 0.001. No significant differences in CRP and calprotectin levels were observed between groups B1 and B2 (Table 2).

**Figure 1:**
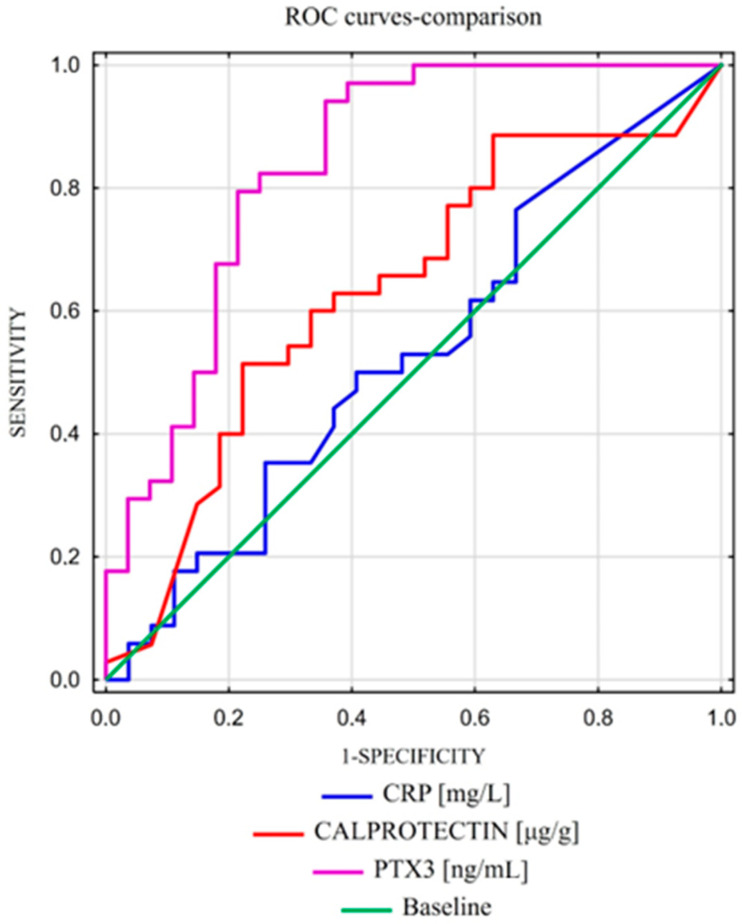
Comparison of PTX3 with other indicators, such as CRP and calprotectin.

The diagnostic accuracy for identifying the fibrotic phase of Crohn’s disease was assessed using the area under the ROC curves. These curves were created by plotting sensitivity against 1—specificity. As far as the area under the curve (AUC) is concerned, a 95% CI (Confidence Interval) was calculated. The optimal cut-off point was determined based on the ROC analysis, selecting the value that maximized the sum of sensitivity and specificity, which is the closest to the top-left corner of the ROC plot. For each optimal cut-off, sensitivity, specificity, and positive predictive value (PPV) were evaluated. The marker for detecting the fibrotic phase (B2) that was considered best was PTX3, with AUCs = 0.839 (Figure 1). The optimal PTX3 cut-off for predicting the fibrotic phase was 3.442 ng/mL, with a sensitivity of 94.10%, specificity of 64.3%, positive predictive value (PPV) of 76.20%, and negative predictive value (NPV) of 90% (Table 3).

ROC curves for CRP and calprotectin values for predicting the fibrosis phase had inferior AUCs of 0.529 and 0.633. We analyzed the diagnostic accuracy of the measured parameters in predicting the fibrosis phase. This was the best for pentraxin 3 and amounted to 80.60%, while CRP and calprotectin amounted to 57.40 and 62.90, respectively (Table 3).

A pairwise assessment of ROC curves was conducted using the DeLong method. To detect the stenotic phenotype B2, PTX3 (AUC = 0.839) showed significantly better performance than CRP (AUC = 0.529, *p* = 0.0044). Additionally, PTX3 was superior to calprotectin for detecting phenotype B2 (AUC = 0.839 vs. AUC = 0.633, *p* = 0.039).

The optimal cut-off-value for PTX3 was determined using ROC analysis, after which the specificity, sensitivity, positive predictive value (PPV), negative predictive value (NPV), and accuracy (ACC) of PTX3 were calculated.

## 3. Discussion

The role of pentraxins, particularly short pentraxins, as transmission factors in the cascade activation of the immune system has been well known. However, to the best of our knowledge, our study is the first to investigate the correlation between the level of PTX3 and what seems to be an even more interesting correlation between PTX3 concentration and fibrosis in the course of CD.

To date, in clinical practice, we have no methods for diagnosing fibrotic strictures in the course of Crohn’s disease other than imaging tests. Determining the exact stage and phenotype of the disease (inflammatory, fibrosing, penetrating) influences the selection of therapy, allowing the prediction of expected end points of the achieved therapeutic effect.

The search for fibrosis markers and the possibility of measuring them with minimally invasive tests is, therefore, key to improving patient care.

PTX3 is one of the molecules acting at various pathogenic stages of IBD. It binds to many ligands, including PAMPs and DAMPs, which activate the TLR-dependent pathway, stimulating the maintenance of the inflammatory process. Moreover, by binding to the components of the extracellular matrix, it participates in the process of pathological tissue remodeling. In the process of proper healing, activated fibroblasts undergo further transformation into myofibroblasts and then apoptosis after achieving the effect of their action. In the course of IBD, impaired fibroblast apoptosis is observed, which leads to the accumulation of active fibroblasts and the development of fibrosis.

What regulates „stricture” fibroblasts remains unclear. The upregulation of the TLR-related pathway, activated by an impaired mucosal barrier, leads to the higher secretion of IL-6 and IL-8. This process seems to be influenced by micro-RNAs and finally results in the stimulation of fibroblast survival and PTX production [22,23].

In our study, we noted higher levels of PTX 3 in the fibrotic phase of Crohn’s disease than in the inflammatory stage and control group. The differences between groups B1/B2 were statistically important with *p* < 0.001 (Table 2). The positive predictive value (PPV) of PTX3 appeared to be similar to calprotectin but higher than CRP, whereas the negative predictive value of PTX3 was significantly higher than both calprotectin and CRP. In our opinion, the presented data showing the level of PTX3 with a cut-off value of 3.442 ng/mL could be considered an effective and efficient model of differentiation between B1/B2 with an accuracy of 80.6%, sensitivity of 94.1%, and specificity of 64.3% (Table 3). The results also confirm that short pentraxins, whose role in monitoring the inflammatory phase in autoimmune disease is substantial in clinical practice, are not efficient markers related to fibrotic CD (ACC 57,4%, sensitivity 66.6%, specificity 33,3%). (Figure 1).

In the literature review, the available studies report the involvement of PTX3 as a modulator of inflammation in inflammatory bowel disease. Based on the conducted research, it was suggested that the measurement of PTX3 could be used as a marker of Crohn’s disease activity. It was also indicated that higher PTX3 concentrations were present in patients with CD compared to patients with UC and healthy individuals [24,25].

However, we have not found any descriptions of the importance of PTX 3 in determining the phenotype of Crohn’s disease, although its connection to tissue remodeling, as well as the usefulness of PTX3 measurements in monitoring the advancement of liver fibrosis, has been demonstrated.

The Narciso-Schiavon et al. studies revealed that the median level of PTX3 in stable cirrhotic patients was significantly higher than that noted in controls (2.6 vs. 1.1 ng/mL; *p* < 0.001) as well as in hospitalized cirrhotic patients compared to stable cirrhotic patients (3.8 vs. 2.6 ng/mL; *p* = 0.001) [26].

In another study on patients with non-alcoholic fatty liver disease (NAFLD), those with NAFLD and fibrosis presented higher plasma levels of PTX3 than both NAFLD patients without fibrosis and the controls (*p* = 0.032 and *p* = 0.028, respectively) [27]. Based on the multivariable analysis of non-invasive biomarkers, including PTX3, the model predicting liver fibrosis, the Pentra score was proposed [28]. However, other published data show a decreasing PTX3 level in late cirrhosis, demonstrating the need for further studies. [29]. The difficulty in interpreting the obtained results may be due to the involvement of pentraxins at different levels of fibrosis advancement.

Nevertheless, the higher levels of PTX3 observed in the group of patients with the stenotic form of Crohn’s disease compared to the inflammatory form in the presented study allows us to hope that it can be considered as a differentiating marker. The specificity of fibrosis in Crohn’s disease interfering with the progressive inflammatory process in other parts of the digestive tract may influence the cut-off points of discriminatory values (B1/B2). It is also necessary to assess the effect of anti-inflammatory treatment; however, it should not affect the level of PTX3 in fixed fibrotic strictures. This research is currently underway.

In fibrotic processes, PTX3 seems to have greater tissue specificity due to its local production by fibroblasts than short pentraxins such as CRP, which is produced mainly in the liver, making PTX3 less dependent on general interference.

In a group of patients with coronary artery disease (CAD), it was shown that higher levels of PTX3 correlate with more advanced CAD and could be postulated as a biomarker of cardiovascular risk before the increase in CRP [30]. In another study, it was also suggested that PTX3, TnT, and CK could predict 3-month mortality in patients, not including liver-derived short pentraxin CRP or NT-pro BNP [31].

As shown in the studies cited above, PTX3 undoubtedly plays a role in controlling the activity of the immune system. Its protective role in maintaining the epithelial barrier, as a site of interaction between the external and internal environment of the body, is crucial and its dysregulation leads to the development of a chronic inflammatory process. Studies assessing the functional state of the epithelial barrier in a group of patients with chronic rhinosinusitis showed that PTX3 was highly expressed in nasal mucosa-derived fibroblasts, contributing to the PTX3 increase [32,33].

By adapting this model to the processes occurring at the level of the intestinal barrier of the gastrointestinal tract, we can verify the role of long pentraxins in the tissue regulation of the immune response as a key compartment in the interaction between the intestinal microbiome and the body’s internal environment. However, in our study, we did not find any relation between PTX3 levels and the clinical activity estimated by the PCDAI scale, which may be connected to the limitation of PCDI itself inflicted, e.g., by the type of Crohn’s disease manifested.

The presented work is an introduction to further assessing the usefulness of PTX3 in monitoring the development of Crohn’s disease, considering the phase and phenotype of the disease.

## 4. Materials and Methods

This study was conducted prospectively on a group of 63 patients with Crohn’s disease aged 5 to 17.5 years old, treated in the Department and Clinic of Pediatrics, Gastroenterology and Nutrition of Wroclaw Medical University, in the years 2022–2023, in whom endoscopic examinations of the gastrointestinal tract and MR enterography were performed due to the suspicion of Crohn’s disease or to monitor the progression of the disease. The diagnoses were made according to the Porto criteria [34]. The Pediatric Crohn’s Disease Activity Index (PCDAI) was used to assess disease activity [35].

In each patient from the study group, the spread and phase of Crohn’s disease were assessed based on the results of endoscopic examinations and magnetic resonance enterography using the Paris scale [36]. The following subgroups were distinguished in the study group: B1—inflammatory—no stenosis or fistulas, and B2—stenotic with the narrowing of the intestinal lumen detected during endoscopic examination and/or magnetic resonance enterography. In the study group, colonoscopies were performed at the time of possessing the serum sample for further evaluation.

To avoid the interference of anti-inflammatory pharmacotherapy with the results of PTX3 measurements, the study included patients in whom any type of biological treatment or systemic steroid therapy was completed at least 2 months before endoscopic examinations/MR enterography (current biological treatment/systemic steroid therapy was a criterion for exclusion from the study), as well as in children with newly diagnosed Crohn’s disease.

The control group consisted of 20 children with functional disorders of the gastrointestinal tract, such as functional dyspepsia, irritable bowel syndrome, and functional constipation, in whom organic disease was excluded during the diagnosis. Characteristics of the study group are presented in Table 1 (Table 1). The purpose of each examination was thoroughly explained, and informed consent was obtained from all participants. The study protocol was approved by the ethics committee of the Bioethics Committee of the 261 Wroclaw Medical University, Poland (no. KB 577/2021, KB 263/2023) and carried out in accordance with the 1975 Declaration of Helsinki (6th, revised in 2008).

Blood samples were collected via a venous puncture in a fasted state on the morning of hospital admission. Serum was extracted from clotted blood (30 min at 25 °C) followed by centrifugation (15 min at 1500× *g*). The separated serum was aliquoted into tubes and frozen at −80 °C for future analysis. C-reactive protein (CRP) and calprotectin levels were measured using typical clinical methods, with data on these levels obtained from patient medical records. PTX3 levels were estimated using the ELISA method (BioVendor Group, Brno, Czech Republic) and following the manufacturer’s protocol. Prior to analysis, the samples were diluted threefold. In BioVendor Human Pentraxin 3 ELISA (Multiscan™ Go microplate reader, Thermo Scientific™, Uusimaa, Finland), standards, quality controls, and samples were incubated in microplate wells pre-coated with polyclonal anti-human PTX3 antibodies. After 60 min of incubation and washing, biotin-labeled polyclonal anti-human PTX3 antibodies were added, followed by a 60 min incubation. After the second wash, the streptavidin-HRP conjugate was introduced. Following a 30 min incubation and final wash, the conjugate reacted with the substrate solution (TMB). The reaction was interrupted by adding an acidic solution. The absorbance of the yellow product of the reaction was measured afterward; it turned out to be directly proportional to the concentration of PTX3. A standard curve was generated by plotting absorbance values against known standard concentrations, which were then used to determine PTX3 levels in unknown samples. The color saturation was measured using an ELISA plate reader (Multiscan™ Go microplate reader, Thermo Scientific™, Uusimaa, Finland). Control samples and serum standards, with concentrations ranging from 78 to 5000 pg/mL, were included in each run. The minimum detectable PTX3 concentration was below 22 pg/mL.

### Statistical Analysis

Nonparametric tests were applied because the measured data did not all follow a normal distribution. To compare the results among patients, the Kruskal–Wallis nonparametric ANOVA test was used for multiple independent trials. A *p*-value of less than 0.05 was deemed statistically significant. The diagnostic value of PTX3 for identifying the fibrotic phase of Crohn’s disease (B2) in comparison to the inflammatory phase (B1) was evaluated by calculating the area under the receiver operating characteristic (ROC) curves. These curves were created by plotting sensitivity against 1—specificity. As far as the area under the curve (AUC) is concerned, a 95% CI (Confidence Interval) was calculated. The optimal cut-off point was determined based on the ROC analysis, selecting the value that maximized the sum of sensitivity and specificity, which was the closest to the top-left corner of the ROC plot. For each optimal cut-off, sensitivity, specificity, and positive predictive value (PPV) were evaluated. All statistical analyses were conducted with the following software: Statistica version 13.3.

## Figures and Tables

**Table 1:** Characteristics of the study group.

Group	Form of the Disease	Paris Scale	Number of Children	Gender		Age (Years)	
				Females	Males	Range	Mean
CD group	Inflammatory	B_1_	28	7	21	9–17.5	14.4
	Scenoti with narrowing	B_2_	35	14	21	5–17	14.6
	Total		63	21	42	5–17.5	14.5
Control group			20	8	12	1–16.5	9.3

CD, Crohn’s disease.

**Table 2:** Associations between PTX3, CRP, and calprotectin Crohn’s disease indices.

Group	Form of the Disease (n)	Paris Scale	PTX3 [ng/mL]	CRP [mg/L]	Calprotectin [μg/g]
CD group	Inflammatory (28)	B_1_	3.06 (1.27–6.99)	2.00 (0.50–45.20)	294 (20.00–2388)
	Scenoti with narrowing (35)	B_2_	4.89 *** (2.98–13.65)	2.55 (0.50–44.00)	1220 (20.0–2740)
Control group	(20)		3.04 (1.01–4.97)	0.5 (0.50–4.30)	-

CD—Crohn’s disease, PTX 3—pentraxin3, and CRP—C reactive protein. Continuous variables are expressed as median (interquartile range; IQR). Significance between group B1 *** *p* < 0.001 vs. group B2.

**Table 3:** PTX3 values for assessing the form of the disease in patients with Crohn’s disease.

	Cut-Off Values	AUC (95%CI)	Sensitivity (%)	Specificity (%)	PPV (%)	NPV (%)	ACC
PTX3 [ng/mL]	3.442	0.839 (0.736–0.843)	94.10	64.3	76.20	90.0	80.60
CRP [mg/L]	0.70	0.529 (0.382–0.677)	66.70	33.3	59.1	52.9	57.40
Calprotectin [μg/g]	1220	0.633 (0.491–0.775)	51.4	77.8	75.0	55.30	62.90

## Data Availability

All relevant data in the current study are available from the corresponding author on request.
